# Inter- and intra-species intercropping of barley cultivars and legume species, as affected by soil phosphorus availability

**DOI:** 10.1007/s11104-017-3365-z

**Published:** 2017-08-08

**Authors:** Tegan Darch, Courtney D. Giles, Martin S. A. Blackwell, Timothy S. George, Lawrie K. Brown, Daniel Menezes-Blackburn, Charles A. Shand, Marc I. Stutter, David G. Lumsdon, Malika M. Mezeli, Renate Wendler, Hao Zhang, Catherine Wearing, Patricia Cooper, Philip M. Haygarth

**Affiliations:** 1 Rothamsted Research, North Wyke, Okehampton, Devon EX20 2SB UK; 2 The James Hutton Institute, AB15 8QH and Dundee, Aberdeen, Scotland DD2 5DA UK; 3 Lancaster Environment Centre, Lancaster University, Lancaster, LA1 4YQ UK

**Keywords:** Legume, Barley, Phosphorus availability, Plant diversity, Yield, Phosphorus uptake

## Abstract

**Aims:**

Intercropping can improve plant yields and soil phosphorus (P) use efficiency. This study compares inter- and intra-species intercropping, and determines whether P uptake and shoot biomass accumulation in intercrops are affected by soil P availability.

**Methods:**

Four barley cultivars (*Hordeum vulgare* L.) and three legume species (*Trifolium subterreneum, Ornithopus sativus* and *Medicago truncatula*) were selected on the basis of their contrasting root exudation and morphological responses to P deficiency. Monocultures and barley-barley and barley-legume intercrops were grown for 6 weeks in a pot trial at very limiting, slightly limiting and excess available soil P. Above-ground biomass and shoot P were measured.

**Results:**

Barley-legume intercrops had 10–70% greater P accumulation and 0–40% greater biomass than monocultures, with the greatest gains occurring at or below the sub-critical P requirement for barley. No benefit of barley-barley intercropping was observed. The plant combination had no significant effect on biomass and P uptake observed in intercropped treatments.

**Conclusions:**

Barley-legume intercropping shows promise for sustainable production systems, especially at low soil P. Gains in biomass and P uptake come from inter- rather than intra-species intercropping, indicating that plant diversity resulted in decreased competition between plants for P.

**Electronic supplementary material:**

The online version of this article (doi:10.1007/s11104-017-3365-z) contains supplementary material, which is available to authorized users.

## Introduction

The finite nature of phosphate ore reserves, their uneven global distribution, and the risk of eutrophication by phosphorus (P) loss to watercourses has created the need to more efficiently use soil P and P containing fertilizers (Steffen et al. 2015; van Dijk et al. 2016). Gains are being made through better management practices, such as improved matching of plant need and supply of fertilizer applications. However, McLaren et al. (2015) demonstrated that the majority of the fertilizer P applied to two Australian pasture soils became sorbed to the soil in inorganic and organic P pools, with only 35% being taken up by clover plants in the year of application. Therefore, there is interest in ways to make the soil P more bioavailable using plant traits (Faucon et al. 2017; Kidd et al. 2016; Wendling et al. 2016), microbial cycling (Richardson and Simpson 20211), and, more recently, intercropping (Xue et al. 2016).

Brooker et al. (2015) define intercropping as ‘two or more crop species or genotypes growing together and coexisting for a time’. Cereal grain and legume intercrops have proven effective for efficient nitrogen (N) use, with a greater total grain production as protein, as well as supressing weeds, pests and diseases, and increasing plant biodiversity (Bedoussac et al. 2015; Brooker et al. 2015). In comparison, research into the benefits of intercropping for P utilization efficiency is in its infancy, but positive effects on biomass and P uptake have been reported (Li et al. 1999; Tang et al. 2016; Xue et al. 2016). These benefits may be due to below-ground complementarity or facilitation effects (Li et al. 2014). Complementarity is a decrease in competition between plants, relative to monocultures. For example, plants may use pools of P not available to the other, or competition may be decreased because plants have different root architectures and explore different soil horizons (Betencourt et al. 2012; Hinsinger et al. 2011). Facilitation occurs when one species makes previously unavailable P available to the other, perhaps due to the exudation of organic acids or phosphatases (Betencourt et al. 2012; Hinsinger et al. 2011), or due to rhizosphere acidification increasing P availability (Li et al. 2008).

For intercropping generally, rather than specifically for a P benefit, it has been shown that the effectiveness of intercropping is dependent on the choice of crops. For example, facilitation between intercropped faba bean (*Vicia faba* L.) and maize (*Zea mays* L.) leads to a positive yield advantage for both crops compared to their monocultures, whereas no yield advantage is measured when intercropping faba bean and wheat due to a lack of facilitation between these species (Wang et al. 2015). Furthermore, greater yields have been measured when the intercropped plants are of different species (inter-species intercropping), rather than different cultivars of the same species (intra-species intercropping)(Schob et al. 2015). However, it is unclear whether this is also true when the measured trait is P uptake, and if so, whether intra-species intercropping can still lead to a biomass and P uptake advantage if the cultivars are selected for maximal diversity. Furthermore, it is unknown whether inter-species intercropping can be affected by the choice of cultivar of one of the plants. These differences may occur due to genetic variability within or between species in their P mobilisation and uptake. This variability may be due to their propensity for root exudates such as organic acids or phosphatases, which directly increase P availability (Kidd et al. 2016), or to exudation of other compounds that increase the microbial biomass, or to different root architectures or arbuscular mycorrhizal inoculation, which result in increased P availability to the plant (Faucon et al. 2015).

Phosphorus availability also influences the effectiveness of intercrops for mobilizing soil P. Root exudation of both acid phosphatases and organic acids is known to be upregulated in plants under limited phosphate availability (Jones 1998; Tadano and Sakai 1991), and therefore greater facilitation between plants may be expected. Indeed, Betencourt et al. (2012) demonstrated that durum wheat (*Triticum aestivum durum* L.)-chickpea (*Cicer arietinum* L.) intercrops had greater rhizosphere soil P availability and plant biomass than the monocultures, and that the proportional increases were greater under no added P conditions than under sufficient P conditions. Generally, the P level of the soil is measured in terms of its phosphate availability. However, if a high proportion of the total P is in organic forms, facilitation may be a more important process (Betencourt et al. 2012).

In this study, we measured the effectiveness of intra- and inter-species intercropping for promoting the utilisation of soil P using four barley cultivars and three legume species with contrasting phenotypic traits. Effect of soil P level was tested in three soil P levels: (i) the original soil, which was initially very P limited and contained the majority of the P in organic forms, then with phosphate added to achieve (ii) slightly limiting and (iii) excess P conditions. The hypotheses tested were that 1) intercropping leads to greater shoot biomass production and P uptake of the intercropped plants relative to the same individuals in monoculture, with a greater effect of inter-species than intra-species intercropping, 2) the barley and legume monocultures with the strongest P acquisition traits lead to the largest gains in P acquisition in the co-cropped plant when in an intercrop, and 3) the P uptake and shoot biomass production in intercrops relative to the monocultures (‘intercropping effectiveness’) are most pronounced under P limiting conditions.

## Materials and methods

### Soil

Topsoil (0–10 cm depth) used for all growth experiments was collected from a continuously managed pasture in Glensaugh, Scotland, UK (56^o^53’42.29″N - 2^o^32’00.42″W). The “Glensaugh” soil is a freely drained podzol (FAO 2014). After collection, the soil was sieved to <10 mm and air-dried, and sieved to <4 mm prior to growth experiments. The soil, selected for its small phosphate concentration, contained 6.7 mg P kg^−1^ Olsen P, and 1.1 mg P kg^−1^ molybdate reactive P and 3.8 mg P kg^−1^ total P in water extracts (1:100 *w*/*v*, 1 h extraction). The degree of phosphorus saturation (DPS) was 10.5%. Further chemical and physical characterisation of the soil is given in Menezes-Blackburn et al. (2016).

### Barley and legume varieties

Barley (*Hordeum vulgare* L.) cultivars and legume species were selected based on the findings of Giles et al. (2017), in which 143 barley cultivars and 6 legume species were screened for exudation characteristics (citrate, phytase, pH change of solution) and root traits (shoot:root, root length, root diameter) after growth in hydroponics and/or sand at low, intermediate and high P (0, 0.5 and 1 mM P as KH_2_PO_4_). Four barley cultivars (Spire, Waggon, Prague and Krystal) and three legume species (*Trifolium subterraneum* [Subterraneum clover], *Ornithopus sativus* [French serradella], and *Medicago truncatula* [Barrel clover]) were selected for intercropping trials on the basis of this earlier study, that showed a wide range of root exudation and morphological responses to P deficiency. Briefly, Prague had low exudation of phytase and citrate, Spire had low phytase but demonstrated exudation of citrate at low P, Krystal had high phytase and showed citrate exudation at high P, and Waggon had phytase and citrate exudation at all P levels. Phosphorus deficiency caused a decrease in exudate pH for Spire, but an increase in exudate pH for Prague and Krystal. The specific root length (m g^−1^ root dry weight) increased in Spire and Prague as a response to P deficiency, but decreased in Waggon and Krystal. Phytase exudation in the legumes was highest in *M. truncatula*, followed by *T. subterreneum* and then *O. sativus*. All plants exuded citrate, but there was no clear trend across the P levels (Giles et al. 2017).

### Critical phosphorus

A critical P test was carried out to ascertain how much P to add to the soil for subcritical and excess P levels. Soil (500 g) was dosed with orthophosphate solution to twelve P levels (0, 50, 100, 150, 200, 250, 300, 400, 500, 600, 700, 1000 mg kg^−1^ added P, on a dry soil weight basis) in duplicate. Soils were potted and pre-incubated in the dark in a controlled temperature room for 1 week. Barley (cv. Optic) seeds were pre-germinated, and 5 seeds were planted in each pot. Throughout the pre-incubation and the growth period, the moisture content of the pots was maintained at 80% of the water holding capacity (equating to 60.5 g water per 100 g soil) using deionised water, and the room temperature was maintained at 21/16 °C day/night (16 h day length), with lighting at 300 W/m^2^. To each pot, 5 mL of nutrient solution (containing 857 mM NH_4_NO_3_, 154 mM KCl, 81 mM MgSO_4_.7H_2_O, 3.3 mM MnCl_2_.4H_2_O, 3.8 mM H_3_BO_3_, 0.1 mM CoSO_4_.7H_2_O, 2.2 mM CuSO_4_.5H_2_O, 1.8 mM ZnSO_4_.7H_2_O and 0.9 mM Na_2_MoO_4_.2H_2_O) was applied in the water at planting, and a further 4.5 mL was applied two weeks after planting. Thereafter, every 7 days 1 mL of a nutrient solution containing 857 mM NH_4_NO_3_ and 77 mM KCl was applied. The plants in one replicate pot from each P level were harvested at 6 weeks, and the others when the grain was ripe, with biomass prepared/analysed as outlined below.

### Experiment 1: Barley-barley intercropping

Experiment 1 was carried out at Rothamsted Research North Wyke, in Okehampton, Devon, UK (latitude 50.77, longitude −3.90) in January 2015. Based on the critical P experiment, 75% of optimal P was determined as an addition of 250 mg kg^−1^ P (Supplementary data Fig. 1). Therefore, to achieve very low, subcritical and excess levels of P, 200 g of soil was dosed with either 0, 250 or 1000 mg kg^−1^ added P. These will subsequently be referred to as P_0_, P_1_ and P_2_. The soils were potted and held at 80% water holding capacity with deionised water. Pots were loosely covered with black plastic, and pre-incubated for 1 week in a greenhouse at 21/16 °C day/night (16 h day length). Afterwards, four barley seeds were planted per pot, with the following treatments: (i) monocultures (4 levels) of the barley cultivars Spire, Waggon, Prague and Krystal; (ii) intercropping (4 levels, with two seeds of each cultivar; hereby termed ‘intra-species intercropping’) of Waggon and Spire, Waggon and Krystal, Spire and Krystal, and Spire and Prague; and (iii) control pots with no seeds. Five replicates pots of each treatment were used at each P level. Total number of pots was 135 ((comprising 4 monocultures +4 intercropped treatments + control) * 3 P levels * 5 reps). Plants were grown at 21/16 °C day/night, with supplementary lighting at a minimum of 200 W/m^2^ outside of natural daylight hours, to maintain a 16 h day length. Eight days after planting, plants were thinned to two per pot, with one plant per cultivar in intercropped treatments, any seeds which failed to germinate were removed. Every 7 days after seed planting, 10 mL of a nutrient solution (containing 52.5 mM NH_4_Cl, 70 mM Ca(NO_3_)_2_, 70 mM KNO_3_, 52.5 mM MgSO_4_.7H_2_O, 0.1 mM FeEDTA, 105 μM MnCl_2_.4H_2_O, 403 μM H_3_BO_3_, 10.5 μM ZnCl_2_, 28 μM CuSO_4_.5H_2_O, Na_2_MoO_4_.2H_2_O and 17.5 μM CoCl_2_, adjusted to pH 5.5 with NaOH) was applied to each pot in the water. The critical P test demonstrated that biomass at six weeks was a good predictor of the final grain biomass (supplementary data, Fig. 1). Therefore, plant biomass was harvested six weeks after seeds were planted.

**Fig. 1:**
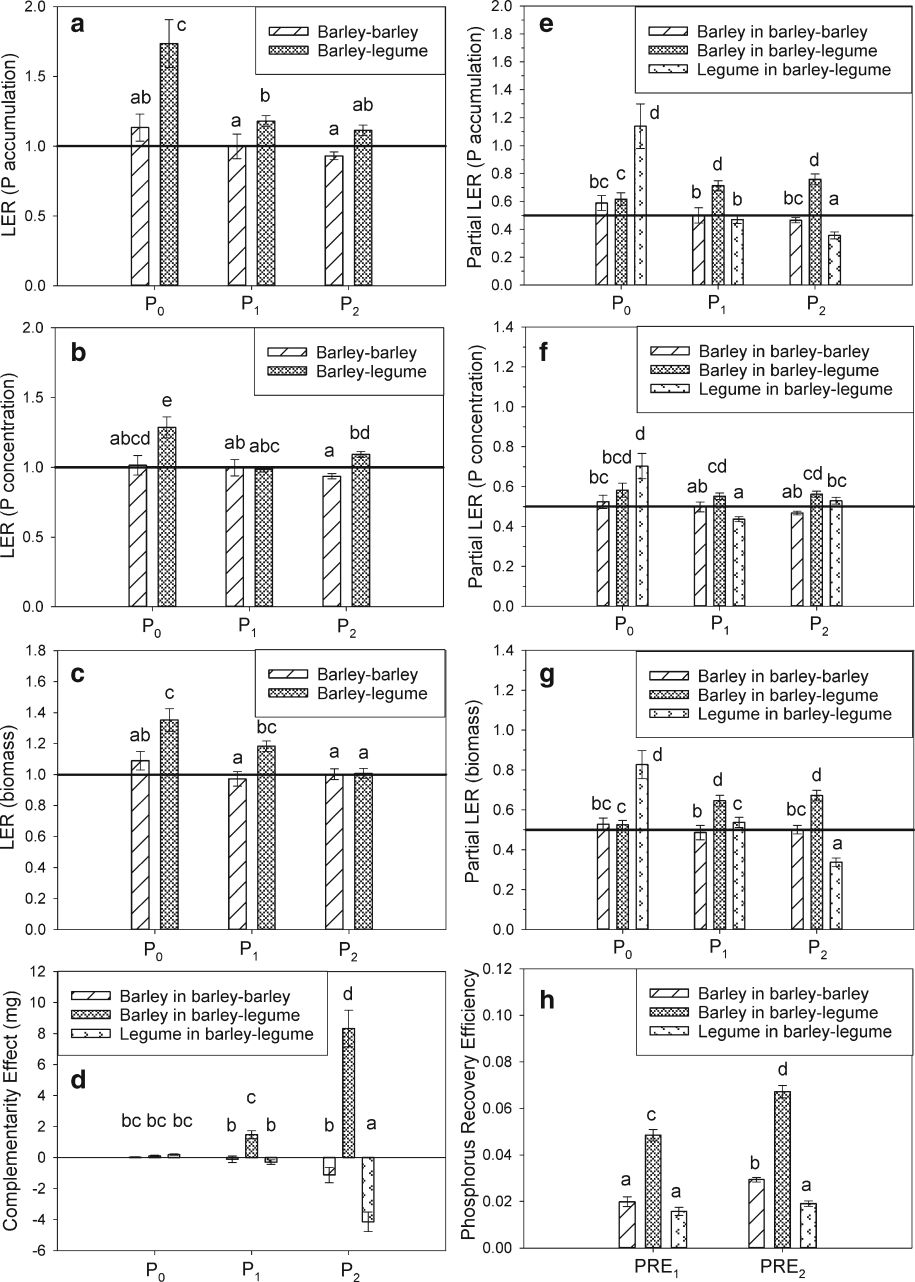
Comparison of the barley-barley and barley-legume intercropping at the three different P levels, P_0_, P_1_ and P_2_ using the metrics **a**) Land Equivalent Ratio (LER) of P accumulation, **b**) LER of P concentration, **c**) LER of biomass, **d**) Complementarity Effect (CE), **e**) partial LER of P accumulation, **f**) partial LER of P concentration, **g**) partial LER of biomass, and **h**) Phosphorus Recovery Efficiency (PRE). Error bars show one standard error of the mean

### Experiment 2: Barley-legume intercropping

Experiment 2 was carried out concurrently with experiment 1 at the James Hutton Institute, Dundee, UK (latitude 56.46, longitude −3.07). Plant treatments were: (i) monocultures (4 levels) of the barley cultivars Spire, Waggon, Prague and Krystal (i.e. duplicating the experiments at Rothamsted, above), (ii) monocultures (3 levels) of legume species, (iii) intercrops of a barley plant and a legume, with all plant combinations trialled (12 levels; hereby termed ‘inter-species intercropping’), or (iv) control pots with no seeds. Total number of pots was 300 ((comprising 4 barley monocultures +3 legume monocultures +12 intercropped treatments + control) * 3 P levels * 5 reps). Seeds were pre-germinated on 1% distilled water agar, and planted two per pot, with one barley and one legume in intercropped treatments. Other procedures and conditions replicated those in experiment 1. However, natural daylight, and hence the hours of supplemental lighting (also at 200 W/m^2^), varied between sites.

### Analyses

For all experiments similarly all above ground material was collected for the analysis of shoot biomass. Dry weight was measured after oven drying to constant mass at 65–70 °C. For the plants grown to yield in the critical P determination, grain was harvested separately to the remainder of the biomass. Phosphorus content of all biomass samples was determined on finely milled material, using a sulphuric acid and hydrogen peroxide digestion (Heffernan 1985), followed by malachite green colourimetry (Irving and McLaughlin 1990) as described previously (George et al. 2011).

### Calculations

Three main metrics were used to determine the effect of intercropping treatments on P uptake and shoot biomass accumulation. In each case, calculations were done with the intercrop and monoculture results from each replicate block, prior to determining the mean of the 5 replicates.

1. Land Equivalent Ratio (LER) is the relative land area required to grow monocultures with the same productivity as the intercrop, and can be calculated for either the individual plants in the intercrop (partial LER), or for the treatment as a whole (He et al. 2013b). We defined productivity as P uptake, and have calculated LERs and partial LERs for both P concentration and P accumulation, given as P in the calculations below.

1 $$ Partial\ {LER}_A=\frac{P\ {intercrop}_A}{P\ {monoculture}_A} $$

2 $$ LER= Partial\ {LER}_A+ Partial\ {LER}_B $$

Where A and B are the two crops in the intercrop treatment. Where the partial LER is >0.5, or the LER is >1, it indicates that intercropping provides an advantage for plant P uptake compared to the monocultures. Where the partial LER is <0.5, or the LER is <1, intercropping is having a detrimental effect on P uptake by plants compared to the monoculture.

2) Complementarity effect (CE) is an absolute measure of the increase or decrease in shoot P accumulation as a result of intercropping and is calculated for each plant in the intercrop treatment:

3 $$ {CE}_A=n\times \Delta {RP}_A\times P\ {accumulation monoculture}_A $$

Where n is the number of species in the intercropped treatment, and ΔRP_A_ is the difference between the observed and expected relative yield for species A (i.e., the difference between the partial LER and a cropping density of 0.5 for an individual plant) (Loreau and Hector 2001).

3) The Phosphorus Recovery Efficiency (PRE) is the amount of P accumulated in the plants relative to the amount added to the soils (Chikowo et al. 2010). Unlike the two previous metrics, PRE is not a direct comparison of the intercropped and monoculture treatments.

4 $$ {PRE}_1=\frac{P\  accumulation\ {P}_1-P\  accumulation\ {P}_0}{250} $$

5 $$ {PRE}_2=\frac{P\  accumulation\ {P}_2-P\  accumulation\ {P}_0}{1000} $$

Where P accumulation P_0,_ P accumulation P_1,_ and P accumulation P_2_ refer to the shoot P accumulation under the P_0_, P_1_ and P_2_ levels of fertilization. The metric PRE_1_ is a comparison between the P_1_ and P_0_ fertilization levels, with 250 mg kg^−1^ added P, while PRE_2_ is a comparison between the P_2_ and P_0_ fertilization levels, with 1000 mg kg^−1^ added P.

Metrics on selection effects and functional diversity were not considered as the growth period was short, the number of plants was controlled, and all above-ground biomass was measured (e.g. Betencourt et al. 2012; Crème et al. 2016; Li et al. 2016).

### Statistical analysis

Statistical analysis was conducted using Genstat (v 18.1.0.17005, VSN International Ltd). Critical P was determined using regression analysis with exponential curve fitting. Two-way ANOVAs of P level and plant species or cultivar, or P level and plant treatment (barley in barley-barley, barley in barley-legume or legume in barley-legume) were used to determine treatment differences based on LER, partial LER, CE and PRE values, and for comparing monoculture shoot P accumulations and concentrations. Significance is reported at *p* < 0.05. Non-normal data were logged before analysis, and post-test determination of differences between treatments was done using the pairwise multiple comparison test ‘amcomparison’.

## Results

### Growth and phosphorus uptake by monocultures

At P_1_ and P_2_ in both the barley-barley and barley-legume experiments, roots pervaded the entire soil volume, whereas at P_0_ roots were less abundant and above-ground growth appeared stunted. There was no nodulation of the legume roots, indicating that plants received sufficient N.

Comparing the P uptake for the legume monocultures, there was a significant interaction (*p* < 0.001) between legume species and P level for shoot P concentration, P accumulation and the PREs. In general, these metrics were greater in *O. sativus* compared to *T. subterreneum* and *M. truncatula*, with a significant effect (*p* < 0.05) of legume species seen at all P levels except for P accumulation at P_2_ (Table 1, Table 2). For example, the mean ± s.e. P concentration in *O. sativus, T. subterreneum* and *M. truncatula* was 2.8 ± 0.14, 1.9 ± 0.11 and 1.4 ± 0.07 mg g^−1^ respectively, while the P accumulation was 2.2 ± 0.20, 0.9 ± 0.15 and 0.6 ± 0.06 mg respectively, both at P_1_ (Table 1). At P_1_, the biomass of *O. sativus* (0.78 ± 0.048 g) was greater (*p* < 0.05) than that of *T. subterreneum* (0.45 ± 0.071 g) and *M. truncatula* (0.43 ± 0.044 g) (Table 1). However, at P_2_ *M. truncatula* had a greater (*p* < 0.05) biomass than *O. sativus* (1.34 ± 0.082 and 0.089 ± 0.143 g respectively)*.*

**Table 1:** Biomass, phosphorus concentration and phosphorus mass of barley and legume monocultures

		P concentration (mg g^−1^)		Biomass (g)		P accumulation (mg)	
		Mean	s.e.	Mean	s.e.	Mean	s.e.
Barley monocultures in barley-barley experiment							
P_0_	Waggon	0.79	0.06 b	0.21	0.03 a	0.17	0.03 b
	Spire	0.85	0.17 b	0.18	0.02 a	0.15	0.04 b
	Prague	1.01	0.29 c	0.20	0.02 a	0.22	0.06 c
	Krystal	0.52	0.23 a	0.16	0.02 a	0.08	0.04 a
P_1_	Waggon	1.27	0.12 c	1.02	0.11 c	1.29	0.16 d
	Spire	1.42	0.08 c	0.78	0.06 b	1.09	0.06 d
	Prague	1.46	0.09 c	0.98	0.08 bc	1.42	0.12 d
	Krystal	1.46	0.13 c	0.92	0.08 bc	1.31	0.08 d
P_2_	Waggon	4.12	0.09 d	1.71	0.04 e	7.05	0.19 e
	Spire	4.64	0.12 d	1.44	0.09 d	6.67	0.43 e
	Prague	4.50	0.28 d	1.50	0.12 d	6.65	0.48 e
	Krystal	3.81	0.09 d	1.61	0.10 de	6.12	0.28 e
Barley monocultures in barley-legume experiment							
P_0_	Waggon	0.74	0.05 a	0.38	0.02 a	0.28	0.02 a
	Spire	0.75	0.06 a	0.37	0.03 a	0.27	0.03 a
	Prague	0.94	0.08 b	0.37	0.03 a	0.34	0.04 a
	Krystal	0.71	0.03 a	0.41	0.02 a	0.29	0.03 a
P_1_	Waggon	1.60	0.09 c	1.46	0.08 c	2.34	0.18 b
	Spire	1.63	0.09 c	1.22	0.09 bc	2.01	0.20 b
	Prague	1.56	0.06 c	1.11	0.06 b	1.73	0.10 b
	Krystal	1.64	0.07 c	1.25	0.13 bc	2.04	0.21 b
P_2_	Waggon	4.92	0.18 d	2.24	0.10 e	11.02	0.64 c
	Spire	4.78	0.12 d	1.99	0.16 de	9.50	0.79 c
	Prague	4.77	0.30 d	1.80	0.23 d	8.46	1.17 c
	Krystal	4.50	0.17 d	2.10	0.16 e	9.49	0.89 c
Legume monocultures in barley-legume experiment							
P_0_	*T. subterreneum*	0.96	0.09 ab	0.21	0.01 ab	0.20	0.02 b
	*M. truncatula*	0.75	0.18 a	0.12	0.02 a	0.08	0.02 a
	*O. sativus*	1.31	0.25 bc	0.16	0.01 ab	0.25	0.06 b
P_1_	*T. subterreneum*	1.90	0.12 de	0.45	0.07 bc	0.87	0.15 c
	*M. truncatula*	1.42	0.07 cd	0.43	0.04 c	0.60	0.06 c
	*O. sativus*	2.76	0.14 e	0.78	0.05 d	2.17	0.20 d
P_2_	*T. subterreneum*	5.01	0.17 fg	1.28	0.07 e	6.39	0.40 e
	*M. truncatula*	4.11	0.26 f	1.34	0.08 e	5.57	0.53 e
	*O. sativus*	7.11	0.23 g	0.89	0.14 d	6.49	1.10 e

Letters show significant differences within columns and within sections (barley monocultures in barley-barley, barley monocultures in barley-legume, legume monocultures in barley-legume), but across all P levels

**Table 2:** Phosphorus Recovery Efficiencies (PREs) in the barley-legume experiment in order of increasing PRE. The PRE of the monoculture or intercrop treatments are given, with the PRE of the two plants combined, and the PRE of the legume only in the barley-legume intercrop. All values have been multiplied by 1000 for ease of reading

Barley cultivar	Legume species	Mean PRE	s.e.
PRE of the two plants combined			
-	*M. truncatula*	38	4 a
-	*T. subterreneum*	41	4 ab
Spire	*M. truncatula*	56	6 abc
Prague	*M. truncatula*	57	6 bcd
Krystal	*M. truncatula*	68	9 cde
Prague	-	68	5 cde
Waggon	*M. truncatula*	69	2 cde
-	*O. sativus*	71	8 cdef
Waggon	*T. subterreneum*	75	5 cdefg
Krystal	*O. sativus*	76	3 defgh
Spire	*T. subterreneum*	76	5 efgh
Krystal	*T. subterreneum*	77	2 efgh
Prague	*T. subterreneum*	78	1 efgh
Spire	-	81	2 efgh
Krystal	-	81	5 efgh
Spire	*O. sativus*	90	2 fgh
Prague	*O. sativus*	91	11 gh
Waggon	*O. sativus*	91	6 gh
Waggon	-	95	7 h
PRE of legume only			
Krystal	*M. truncatula*	8	2 a
Waggon	*M. truncatula*	10	1 ab
Waggon	*T. subterreneum*	10	2 ab
Prague	*M. truncatula*	11.	1 ab
Spire	*M. truncatula*	13	2 abc
Krystal	*T. subterreneum*	14	1 abcd
Waggon	*O. sativus*	18	2 bcd
Prague	*T. subterreneum*	20	3 cd
Spire	*T. subterreneum*	22	2 de
Krystal	*O. sativus*	22	4 de
Prague	*O. sativus*	28	2 ef
Spire	*O. sativus*	32	5 f

Letters show significant differences between and within monocultures and intercrops for the barley and legume combined, or between legume species for the legume only

In barley, there were few significant differences between the cultivars for the growth and P uptake metrics used. However, significant differences (*p* < 0.05) were seen between barley cultivars at P_0_ in the barley-barley experiment, with the P accumulation and P concentration of cultivars in the order Prague > Spire and Waggon > Krystal (Table 1). For example, the P accumulation of Prague, Spire, Waggon and Krystal were 0.22 ± 0.063, 0.15 ± 0.035, 0.17 ± 0.025 and 0.08 ± 0.035 mg, respectively. The biomass of Waggon was greater (*p* < 0.05) than that of Spire at P_1_, and of Spire and Prague at P_2_. For the barley cultivars in the barley-legume experiment, the P_0_ shoot P concentration was greater (*p* < 0.05) in Prague (0.94 mg g^−1^) than in the other cultivars (0.71–0.75 mg g^−1^) (Table 1). Waggon had a greater (*p* < 0.05) biomass than Prague at P_1_ and P_2_. Across all cultivars, a two-way ANOVA between P level and intercropping experiment indicated that barley monocultures in the barley-legume experiment had a significantly greater biomass (*p* < 0.001, 23–49% greater mean P across the P levels) and P accumulation (*p* < 0.001, 31–41% greater mean P) than the barley monocultures in the barley-barley experiment, with the greatest differences under P limitation (data not shown).

### Influence of plant cultivar or species on intercropping effectiveness

The metrics investigating P uptake or biomass for the two intercropped plants combined were generally not significantly affected (*p* > 0.05) by the plant combination – i.e. the selection of barley cultivar or legume species – for either the barley-barley or barley-legume experiments (Table 2). The exception was for the barley-legume experiment, where the PREs of the Spire-, Prague- and Waggon-*O. sativus* intercrops were greater (*p* < 0.05) than the PREs of the Spire-, Prague- and Waggon-*M. truncatula* intercrops. Typically, the PREs of the intercropped treatments were intermediate of the two monocultures (Table 2). However, intercrops of other barley cultivars with *O. sativus* had greater PRE values than for either the barley or the *O. sativus* monocultures, and this difference was significant (*p* < 0.05) in the case of Prague-*O. sativus*, where the intercrop had a PRE of 0.091, but the Prague and *O. sativus* monocultures had PREs of 0.068 and 0.071 respectively (Table 2).

When PREs were calculated for the legume and barley separately, rather than a combined figure for the intercrop, barley cultivars had greater (*p* < 0.001) PREs than the legume species. The PRE_2_ of barley cultivars in the barley-legume experiment was 0.067, relative to 0.019 for the legume species. Thus, the barley cultivar significantly (*p* < 0.05) affected the PREs of *O. sativus* and *T. subterreneum* (Table 2). For example, the PRE of *T. subterreneum* was greater (*p* < 0.05) when intercropped with either Spire or Prague, at 0.022 and 0.020 respectively, than when intercropped with Waggon, at 0.010. The PRE of *O. sativus* intercropped with Spire (0.032) was greater (*p* < 0.05) than when intercropped with Krystal (0.022) or Waggon (0.018), and *O. sativus* intercropped with Prague had a greater (*p* < 0.05) PRE (at 0.028) than with Waggon (0.018).

### Effect of soil phosphorus level

Due to the absence of significant differences in intercropping effectiveness as a result of the choice of barley or legume, the plant combinations have been combined from this point forward. In the barley-barley experiment, the LERs for the two plants combined were only significantly different (*p* < 0.05) from 1 for P accumulation and P concentration at P_2_, where P uptake was lower in the intercrop than in the monocultures (Fig. 1a–c). In the barley-legume experiment, the LERs ranged from 1.1–1.7 for P accumulation (representing a 10–70% increase in P accumulation), 1.0–1.3 for P concentration (0–30% increase), and 1.0–1.4 for biomass (0–40% increase); generally, the values were significantly greater than 1 (*p* < 0.05). The LERs were greatest at P_0_ in the barley-legume experiment.

Partial LERs indicate the effect of intercropping on the individual crops in the system, with a value greater than 0.5 meaning that the intercrop is more effective than the monoculture. In the barley-barley experiment, the partial LER was calculated for all of the barley combinations, and therefore show the same pattern as the LER (Fig. 1). Across the soil P levels, the barley in the barley-legume experiment displayed a positive effect of intercropping, with partial LERs of 0.62–0.76 for P accumulation, 0.55–0.58 for P concentration, and 0.53–0.67 for biomass (Fig. 1 e, f, g). The increase in partial LER for barley in the barley-legume experiment was significant for P accumulation (*p* < 0.05) and biomass (*p* < 0.001).. For the legumes in the barley-legume experiment, the partial LERs had a different pattern, being highly positive at P_0_ and then decreasing (*p* < 0.001) with P level, to a value below 0.5. For example, the partial LER of legume biomass decreased from 0.83 to 0.34, and P accumulation decreased from 1.14 to 0.36.

The CE metric indicates the absolute change in P content of each plant in a combination relative to the monoculture. The pattern across the P levels was the same as for the LERs and partial LERs (Fig. 1d). However, the gain or loss in P tended to be magnified at P_2_ due to the greater size of the plants. In the barley-barley experiment, there was no significant change (*p* > 0.05) in CE across the P levels (supplementary information). For barley in the barley-legume experiment, the CE increased with P level, and was greater (*p* < 0.05) at P_2_ than P_0_ and P_1_, with increases of 8.32 mg per plant at P_2_ above the monoculture P of 8.46–11.02 mg. Barley in the barley-barley and barley-legume experiments had greater (*p* < 0.05) PRE_2_s than PRE_1_s (Fig. 1h). In contrast, the legume in the barley-legume experiment had a decrease in CE with P level, and was lower (*p* < 0.05) at P_2_ than at P_0_ or P_1_. At P_2_, the legumes had 4.15 mg less P than the monocultures, relative to monoculture P of 5.57–6.49 mg.

### Comparison of inter- and intra-species intercropping and plant type

The LERs of P accumulation, P concentration and biomass for the inter-species (barley-legume) experiment were always greater (*p* < 0.05) than for the intra-species (barley-barley) experiment at P_0_ (Fig. 1a–). For example, the LERs show that barley-legume intercrops had a 35% and a 74% increase in biomass and P accumulation respectively at P_0_, compared to a 9% and a 13% increase for barley-barley. Furthermore, the partial LERs of P accumulation, P concentration and biomass of the barley in the barley-legume experiment were greater (*p* < 0.05) than for the barley-barley experiment at P_1_ and P_2_ (Fig. 1e–g). For instance, at the partial LERs for biomass and P accumulation at P_2_ were 0.67 and 0.76 (17% and 26% increase relative to the monocultures) for barley in the barley-legume experiment, and 0.50 and 0.47 (0% and 4% decrease relative to the monocultures) for the barley-barley experiment.

At P_0_, the legume had greater (*p* < 0.05) partial LERs of biomass and P accumulation than the barley (Fig. 1a, c), with a 33% increase in legume biomass compared to the monocultures, but only a 3% increase in barley biomass. However, at P_1_ and P_2_, the partial LERs for biomass and P accumulation were greater (*p* < 0.05) for the barley than the legume, showing a 17% increase in biomass for the barley but a 16% decrease for the legumes. Only at P_1_ was there a significant difference (*p* < 0.05) between the two plants for their partial LERs of P concentration, with barley having a greater value than the legume. In the barley-legume experiment, the barley had a greater (*p* < 0.05) PRE_1_ and PRE_2_, and CE at P_1_ and P_2_, than the legume (Fig. 1d, h), with a change in CE relative to the monoculture of 8.3 mg for barley but −4.1 mg for the legumes at P_2_. This is consistent with the greater PRE values of barley monocultures in comparison to the legumes (Table 2; supplementary info).

## Discussion

### Plant diversity

It was hypothesised that diversity in plant traits is an important driver of biomass and P uptake, and this study tested the effect of both genetic (intra-species) and inter-species diversity. The barley-barley intercropping resulted in no significant gain in biomass, P concentration or P accumulation in the intercropped treatments compared to the monocultures. This indicates that neither facilitation nor complementarity occurred in plant treatments containing the same species. Indeed, the P uptake was significantly reduced at the greatest soil P level in this experiment relative to the monocultures, indicating that there was increased competition for P between the intercropped cultivars compared with the monoculture plants. In contrast, the barley-legume intercropping had a positive LER for P accumulation, P concentration and biomass across the P levels, when the effect on both plants was considered together. It is possible that complementarity, where the plants use pools of P not available to the other (Hinsinger et al. 2011), is important. Complementary P use between wheat and legumes was reported by Nuruzzaman et al. (2005) where a wheat crop was preceded by either another wheat crop, a legume crop (either lupin (*Lupinus albus* L.), field pea (*Pisum sativum* L.) or faba bean) or no crop. The wheat shoot yield and P concentration were not significantly different in the latter two treatments, but were significantly different from the treatment where wheat crops followed one another. This implies either that legumes are able to increase P availability to an extent that counterbalances their P use, or that they are solely using forms of P not available to wheat, or a combination of the two (Nuruzzaman et al. 2005). Similar effects have been reported in studies where the crops are grown concurrently (Li et al. 2016).

Our results are the first to show a greater effect of inter-species diversity (barley-legume) than intra-species diversity (barley-barley) on P uptake in intercropping systems. Previous studies have demonstrated comparable results for biomass production. In a comparison of 5 barley cultivars or 5 weed species, inter-species diversity had 8 times the effect on biomass production than did intra-species diversity, with the latter only having weak complementary effects (Schob et al. 2015). This effect was a result of the greater disparity between the weed species than between the barley cultivars grown in a monoculture, for growth traits such as biomass, plant height, leaf area and carbon (C) and nitrogen (N) content (Schob et al. 2015). Similarly, genetic diversity within grass and legume species displayed no effect on biomass production under drought or irrigated conditions, but an effect was seen due to species diversity under drought conditions (Prieto et al. 2015).

### Successful monocultures do not benefit co-cropped plants

The barley cultivars and legume species used in this study were those shown in a previous study (Giles et al. 2017) to be most responsive to P deficiency, and that had the most contrasting exudate and root characteristics (within a plant type), after growth in hydroponics and/or sand. However, plants can change their exudation and root characteristics through physiological plasticity according to soil conditions to affect soil P utilization (Zhang et al. 2016). Consequently, differences between monocultures in their ‘success’ was assessed according to their P uptake and yield metrics, and it was hypothesised that the monocultures most successful in P acquisition would lead to the largest gains in shoot P and biomass in the co-cropped plant when intercropped. For the barley cultivars, differences in monoculture yield and P uptake metrics were often not significant across the P levels, or significant differences were not consistent across the different metrics used. In contrast, *O. sativus* had significantly larger PREs, P accumulation and P concentrations than *M. truncatua* and *T. subterreneum.* Similar relative legume productivities have previously been reported (de Ruiter 1981).

To the authors’ knowledge, this is the first time intercropping studies have compared monocultures for P uptake and biomass, and looked at the relative success of these monocultures in inter- and intra-species intercropping, and therefore there are no comparable data. However, there are a number of reasons why the legume most successful in obtaining P and gaining shoot biomass, *O. sativus*, may not have resulted in significantly better yield and P uptake in the intercropped barley than did *M. truncatula* and *T. subterreneum*. The success of the *O. sativus* monoculture is likely to be due to a mixture of root trait and exudate effects. Root traits which enable plants to scavenge P more effectively are those which increase the surface area, either through an increased root to shoot ratio, increased root length or amount, and more numerous fine roots or root hairs (Kidd et al. 2016). These root traits will only be advantageous to the co-cropped plant if they result in additional root intermingling, and hence a greater chance of facilitation. However, this trait is unlikely to be a factor in a potted study where roots pervaded the whole of the soil volume (Hinsinger et al. 2011). Furthermore, if root growth was restricted in our pot study, it may have negated any potential differences in root morphology between plants. Consequently, any root traits which cause *O. sativus* to scavenge more P than the other two legumes (e.g., deep roots) only benefit the *O. sativus* and not the intercropped barley; any complementarity between the legume and barley is thus irrespective of the legume species. More commonly, the barley would be expected to benefit through facilitation (Hinsinger et al. 2011), where exudates from the legume solubilise and mineralise organic P, which is then available for use by the barley. However, plant exudates only diffuse short distances in the soil (Hinsinger 2001), and may become sorbed to the soil or degraded by the soil microbes for their C content (George et al. 2005; Jones 1998). Furthermore, any P released may be utilised by the soil microbes, especially as the pool of soil microbes (which were not measured in our soils) is often greater under intercrops than under monocultures (Li et al. 2010; Tang et al. 2014). As a result, differences in exudation between *O. sativus* and the other two legumes may be too small to significantly affect P uptake and plant growth of the intercropped barley in a pot trial. In a field trial, root morphology may be more important, increasing the potential for complementarity between plants. On the other hand, the likelihood of facilitation occurring is lower, due to the decreased intermingling of plant roots. Therefore, further work is required to determine whether the legume species would affect intercropping effectiveness at the field scale.

### Intercropping effectiveness most pronounced under P limiting conditions

It was hypothesised that gains in plant growth and shoot P accumulation through intercropping would be greatest under P limiting conditions due to the stress-gradient hypothesis, which suggests that plant complementarity and facilitation are maximised under challenging conditions (He et al. 2013a). For example, root exudation of organic acids, acid phosphatase and phytase tend to be greater under P deficient conditions (Hayes et al. 1999; Jones 1998; Tadano and Sakai 1991). In a durum wheat-chickpea intercrop, an increase in shoot and root biomass of the durum wheat was recorded in a limited P availability soil, but not in an adequate P availability soil. The authors attribute this increase to facilitation via root induced alkalisation (Betencourt et al. 2012), and barley is also known to alkalize their environment (Giles et al. 2017). However, the stress-gradient hypothesis does not always hold true. A durum wheat-faba bean intercrop at very low, low and high soil P had no significant differences in LERs for shoot biomass and P uptake, and partial LERs were generally greater than 0.5 (Tang et al. 2016). This may be due to the greater root biomass at greater P, leading to increased complementarity, and greater root intermingling, leading to increased facilitation. It has also been noted that two similarly stress-tolerant species are likely to be competitive under resource stress, rather than facilitative (He et al. 2013b; Maestre et al. 2009). In our results, the barley-legume intercrop LERs for biomass and P accumulation showed that the intercropping effectiveness was significantly greater under very limiting P conditions than at the slightly limiting or excess P levels. This observation appears to agree with the stress-gradient hypothesis. On the other hand, the differing effect of P level on the two crops – with the partial LERs of biomass and P accumulation for barley increasing with P level, and those for the legume decreasing to negative values – suggest that the LER is merely a balance of the competitiveness of the two crops, which changes with P level.

A change in the relative productivity of the barley and legume across the different P levels could have consequences for the overall intercropping effectiveness, given the smaller biomass and P content of the legumes compared to the barley plants. Such selection effects have been seen in a P sufficient soil in interspecific weed mixtures, where a productive plant out-competed other plants and had a knock-on effect on the productivity of the mixture as a whole (Schob et al. 2015). Furthermore, an effect of P level on the selection effects has been observed in grass-legume intercrops. With limited soil P, the grass *Festuca arundinacea* comprised 15% of the total P harvested and 13% of the total yield when intercropped with the legume *Lotus tenuis*, but at greater P availability it was more competitive, with 42% of the total P and 34% of the yield (Mendoza et al. 2016). In our study, the plants were N fertilized, but it is probable that if this were not the case that the legume would have been more competitive at greater P levels, as measured in ryegrass-white clover intercrops (Høgh-Jensen and Schjoerring 2010). In addition to N status, there are two further factors which may affect the relative competitiveness of the barley and legume plants. Firstly, the soil P levels were determined through a critical P assessment for barley only, and so the terms very limiting/limiting and excess soil P may not apply to the legume as well. However, an assessment of the critical P for 12 legumes, including *T. subterreneum* and another *Medicago* species, found values in the same order of magnitude as we determined for barley (Moir et al. 2016), so this is unlikely to be a substantial factor in our results. Secondly, the greater height and size of the barley compared with the legumes could have caused shading of the latter, and is likely to have reduced the biomass of the legume (Su et al. 2014).

## Conclusion

Previous research has shown that intercropping can result in greater yields and P uptake in crops, and our data suggest that barley-legume intercropping is potentially another combination where this is feasible. By allowing greater utilisation of soil P, and with a concomitant decrease in P fertilizer application, barley-legume intercrops could play a role in sustainable crop production. Furthermore, as increased N use efficiency and the transfer of atmospheric N to soil has also been measured in legume-cereal intercrops, the intercropping of barley and legumes is likely to have numerous benefits. Soil P level is an important control on the relative competitiveness of crops in barley-legume intercrops, with barley increasing in competitiveness with an increase in soil P in a N-sufficient system. On the other hand, increasing diversity in a barley crop by promoting mixtures of barley genotypes does not appear to be an effective approach to improving soil P use efficiency. The difference between the barley-barley and barley-legume intercrops implies that complementarity is important for improving biomass and P uptake in intercrops relative to the monocultures. In summary, barley-legume intercropping shows promise, but needs to be trialled in a field system before recommendations can be made about its viability in an agricultural system.

## Electronic supplementary material

ESM 1 Fig. 1. Barley shoot and grain biomass at the 6-week growth and ripe grain stages, and calculation of the critical P (added P in mg/kg soil) at between 75% and 98% of the maximum yield. (JPEG 153 kb)

ESM 2 Table 1. Shoot biomass, shoot P concentration, shoot P accumulation, Land Equivalent Ratios (LERs) and partial LERs of biomass, P concentration and P accumulation, Complementarity Effect (CE) and Phosphorus Recovery Efficiency (PRE) data for each monoculture and intercrop in the barley-barley and the barley-legume intercropping experiments. Data are the mean and standard error of five replicates. Results of the 2 way ANOVAs (plant and P level) for comparisons between monoculture biomass, P concentration and P accumulation, and 2 way ANOVAs (plant treatment and P level) between LERs and partial LERs of biomass, P concentration and P accumulation, and CEs, for the barley-barley and barley-legume experiments. (XLSX 49 kb)

## Abbreviations

LER: Land equivalent ratio
CE: Complementarity effect
PRE: Phosphorus recovery efficiency
C: Complementarity
F: Facilitation
I: Intercropping
