# Supplementary figures and images for: Inter- and intra-species intercropping of barley cultivars and legume species, as affected by soil phosphorus availability
Source: Plant Soil. 2017 Aug 8;427(1):125–38. doi: 10.1007/s11104-017-3365-z (PMC6438642; doi:10.1007/s11104-017-3365-z)

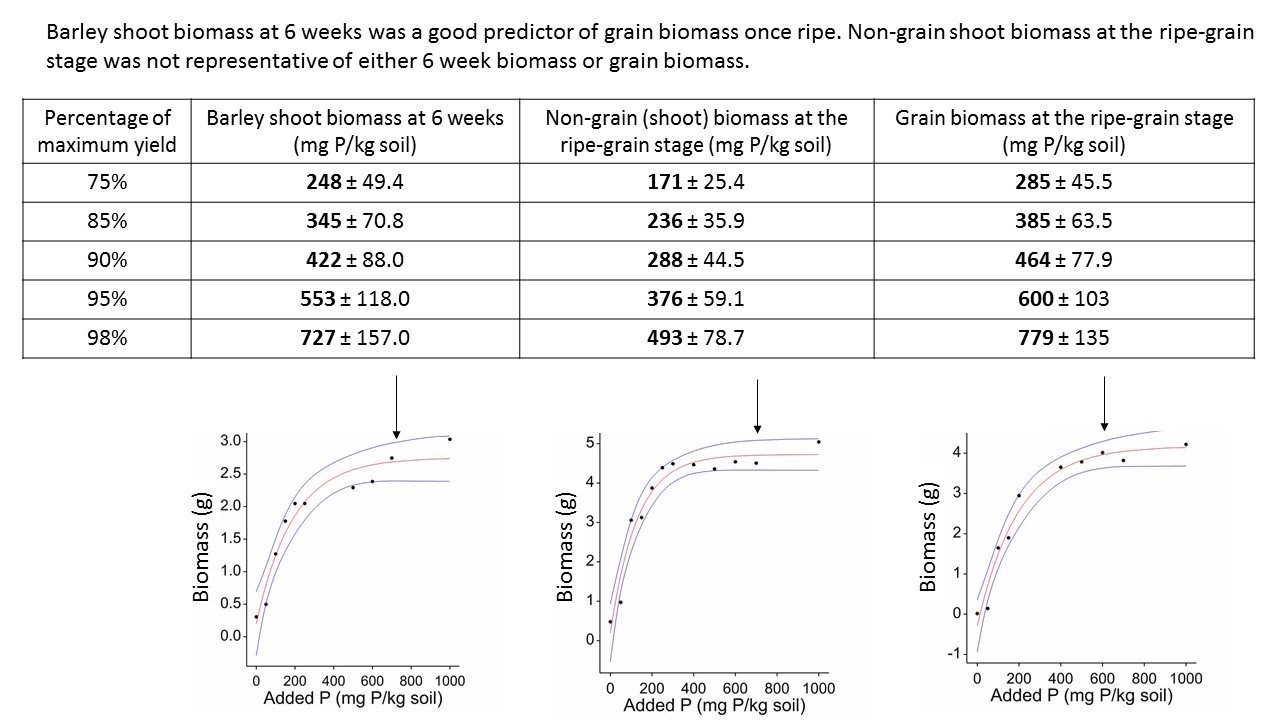

Supplement: Supplementary file 1 — Fig. 1. Barley shoot and grain biomass at the 6-week growth and ripe grain stages, and calculation of the critical P (added P in mg/kg soil) at between 75% and 98% of the maximum yield. (JPEG 153 kb) [file 11104_2017_3365_MOESM1_ESM.jpg]
